# Argument-predicate distance as a filter for enhancing precision in extracting predications on the genetic etiology of disease

**DOI:** 10.1186/1471-2105-7-291

**Published:** 2006-06-08

**Authors:** Marco Masseroli, Halil Kilicoglu, François-Michel Lang, Thomas C Rindflesch

**Affiliations:** 1 Bioengineering Department, Politecnico di Milano, Milan, Italy; 2 Lister Hill National Center for Biomedical Communications, National Library of Medicine, Bethesda, USA

## Abstract

**Background:**

Genomic functional information is valuable for biomedical research. However, such information frequently needs to be extracted from the scientific literature and structured in order to be exploited by automatic systems. Natural language processing is increasingly used for this purpose although it inherently involves errors. A postprocessing strategy that selects relations most likely to be correct is proposed and evaluated on the output of *SemGen*, a system that extracts semantic predications on the etiology of genetic diseases. Based on the number of intervening phrases between an argument and its predicate, we defined a heuristic strategy to filter the extracted semantic relations according to their likelihood of being correct. We also applied this strategy to relations identified with co-occurrence processing. Finally, we exploited postprocessed *SemGen *predications to investigate the genetic basis of Parkinson's disease.

**Results:**

The filtering procedure for increased precision is based on the intuition that arguments which occur close to their predicate are easier to identify than those at a distance. For example, if gene-gene relations are filtered for arguments at a distance of 1 phrase from the predicate, precision increases from 41.95% (baseline) to 70.75%. Since this proximity filtering is based on syntactic structure, applying it to the results of co-occurrence processing is useful, but not as effective as when applied to the output of natural language processing.

In an effort to exploit *SemGen *predications on the etiology of disease after increasing precision with postprocessing, a gene list was derived from extracted information enhanced with postprocessing filtering and was automatically annotated with *GFINDer*, a Web application that dynamically retrieves functional and phenotypic information from structured biomolecular resources. Two of the genes in this list are likely relevant to Parkinson's disease but are not associated with this disease in several important databases on genetic disorders.

**Conclusion:**

Information based on the proximity postprocessing method we suggest is of sufficient quality to be profitably used for subsequent applications aimed at uncovering new biomedical knowledge. Although proximity filtering is only marginally effective for enhancing the precision of relations extracted with co-occurrence processing, it is likely to benefit methods based, even partially, on syntactic structure, regardless of the relation.

## Background

### SemGen

In an effort to minimize errors due to natural language processing (NLP), we developed and evaluated a procedure for postprocessing extracted genetic information. This processing is applied to the output of *SemGen *(Semantics for Genetics), an NLP system for extracting semantic predications (or relations) from the text of Medline citations [1,2].

*SemGen *applies in the domain of molecular genetics and has several components: Journal Descriptor Indexing (JDI) [3], the MedPost tagger [4], the SPECIALIST Lexicon [5], the UMLS Metathesaurus [6], MetaMap [7], and ABGene [8]. These components interact to first identify genetic phenomena and disorders and subsequently construct semantic relations among these entities: gene-gene interactions (STIMULATE, INHIBIT, INTERACT_WITH, and their negations) as well as gene-disease associations (ASSOCIATED_WITH, PREDISPOSE, CAUSE, and their negations).

**Figure 1:**
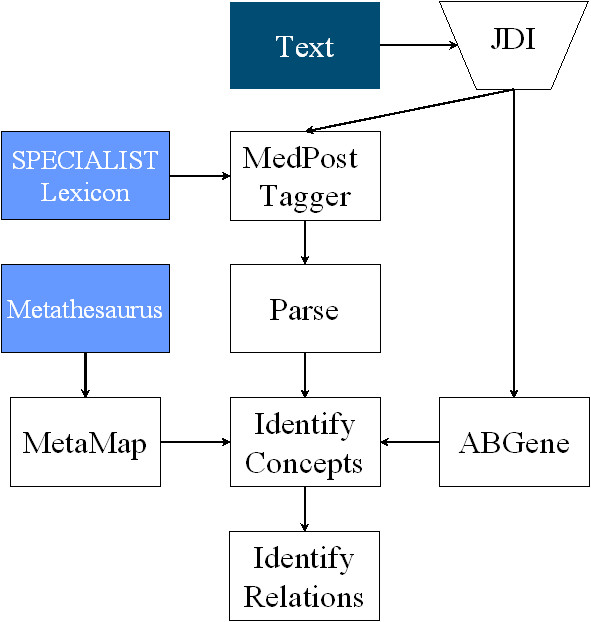
**SemGen components and workflow**. JDI: Journal Descriptor Index.

Processing consists of three phases: construction of an underspecified syntactic structure identification of relevant semantic concepts, and final interpretation of a semantic predication. The system first calls JDI, a statistics-based labeled categorizer used to limit input text to the molecular genetics domain before proceeding with natural language processing. After JDI has applied, text is sent both to the MedPost tagger and to ABGene, which assists in identifying gene names (Figure 1).

As the first step in creating an underspecified syntactic structure, the MedPost tagger, drawing on the SPECIALIST Lexicon, labels the words in input (1) with part-of-speech categories, as shown in (2). (Abbreviations include: pron for pronoun, adv for adverb, compl for complementizer, conj for conjunction, aux for auxiliary, adj for adjective, and prep for preposition.)

(1) We now show that apoA-II promotes insulin resistance and has diverse effects on fat homeostasis

(2) pron(we) adv(now) verb(show) compl(that) noun(apoA-II) verb(promotes) noun(insulin) noun(resistance) conj(and) aux(has) adj(diverse) noun(effects) prep(on) noun(fat) noun(homeostasis)

The tagged list in (2) serves as input to an underspecified (or shallow) parser which identifies phrases in an input string. These correspond to low level nodes in a syntactic parse tree, and except for noun phrases (NP), are left unlabeled. In addition, words are assigned their role inside the noun phrase, as either modifier (mod) or head. Informally, a head is the most important word in a noun phrase. A schematic example is given in (3), where phrases are delimited by square brackets.

(3) [[pron(we)] [adv(now)] [verb(show)] [compl(that)] [head(apoA-II)]_NP _[verb(promotes)] [mod(insulin) head(resistance)]_NP _[conj(and)] [aux(has)] [mod(diverse) head(effects)]_NP _[prep(on) mod(fat) head(homeostasis)]_NP_]

The syntactic structure in (3) serves as the basis for the next phase, identification of noun phrases expressing relevant semantic concepts: genetic phenomena and disorders. Genetic phenomena are defined broadly as those concepts that may have a bearing on molecular genetics and include genes, proteins, nucleotide sequences, mutations, polymorphisms, and chromosomes. In this study we concentrated exclusively on genes. For each gene name identified, *SemGen *attempts to provide the corresponding official symbol and Entrez Gene ID, although this is not always possible. Gene symbol resolution is a challenging NLP task, currently under active investigation (for example Morgan et al. 2004 [9], Hou and Chen 2004 [10], Yu et al. 2002 [11]). For disorders, *SemGen *considers a concept having one of the following UMLS semantic types to be relevant: 'Pathologic Function', 'Disease or Syndrome', 'Neoplastic Process', and 'Congenital Abnormality'.

During processing to identify relevant semantic concepts, *SemGen *examines each noun phrase in structures such as (3) to determine whether it qualifies as a genetic phenomenon or as a disorder. For disorder names, MetaMap is used exclusively to determine whether the phrase maps to a concept in the UMLS Metathesaurus having a relevant semantic type. In considering genetic phenomena, *SemGen *first calls on MetaMap and the UMLS Metathesaurus; however, since the Metathesaurus is not complete for gene names, the output from ABGene is also consulted.

ABGene identifies gene and protein names in text using both statistical and empirical methods. Strategies include low frequency trigrams and rules generated both automatically and by hand. In addition, the program relies on linguistic information, such as cue words, suffixes, and part-of-speech information. *SemGen *directs ABGene to identify all gene names in the Medline citation that contains the sentence currently being processed. These are then available as a resource to *SemGen*. For example, ABGene identifies the gene names in (4) from a citation (PMID 15635645) with title *Structural variants in the retinoid receptor genes in patients with schizophrenia and other psychiatric diseases*.

(4) "NURR1 gene", "RARs", "RAR/RXR", "Retinoid receptors", "retinoid receptor genes", "RXRgamma", "RXR genes"

In processing input from this citation that contains the phrase *NURR1 gene*, *SemGen *determines from MetaMap that *NURR1 *does not occur in the Metathesaurus. The ABGene list (4) for the citation containing the current input sentence is then consulted, and *NURR1 *is successfully identified as a genetic phenomenon in the sentence containing this phrase.

The structure in (5) is an example of the syntactic parse (3) enhanced with identification of a gene and a disorder. The phrase containing "apoA-II" has now been expanded to include the Entrez Gene ID, official symbol, gene name isolated by *SemGen *as well as the original text. The noun phrase containing "insulin resistance" has been expanded to include the UMLS concept, (with semantic type 'Pathologic Function') as well as the original text.

(5) [[pron(we)] [adv(now)] [verb(show)] [compl(that)] [genphenom(336|APOA2|apoa-ii|noun(apoA-II)]_NP _[verb(promotes)] [disorder(mod(insulin) head(resistance)|Insulin Resistance)]_NP _[conj(and)] [aux(has)] [mod(diverse) head(effects)]_NP _[prep(on) mod(fat) head(homeostasis)]_NP_]

In identifying relations, the final phase of processing, *SemGen *relies on concepts identified in the previous phase, some of which serve as arguments of predications. *Indicators *constitute a crucial aspect of this phase. These are syntactic elements that map to the predicate of a semantic predication. Verbs commonly serve this function. For example, in (6), the verb "promote" indicates the semantic predicate predispose. This forms the basis for construction of a predication with arguments "JNK" and "diabetes."

(6) There appear to be multiple mechanisms through which JNK might **promote **diabetes.

Other examples of verbs that indicate semantic relations between a gene and a disorder include "predispose" (for PREDISPOSE) and "influence" and "implicate" (for ASSOCIATED_WITH). Similarly, verbs indicating a relation between two genes are "stimulate" and "upregulate" (for STIMULATE), "block" and "inhibit" (for INHIBIT), and "mediate" (for INTERACT_WITH).

In addition to verbs, prepositions may serve as indicators of semantic predicates. For example, in (7), the preposition "in" indicates the predicate ASSOCIATED_WITH (having arguments "LRRK2" and "Parkinson disease").

(7) A clinic-based study of the LRRK2 gene **in **Parkinson disease yields new mutations.

The preposition "for" can also indicate ASSOCIATED_WITH, as in (8).

(8) Saitohin represents a candidate gene **for **Parkinson's disease.

It should be noted that in both (7) and (8) the preposition is the only syntactic cue that there is a semantic relation between the gene and the disorder. In the molecular genetics domain, we have encountered prepositions as indicators only for the semantic predicate ASSOCIATED_WITH.

*SemGen *has rules that encode indicators for the semantic predicates relevant to this domain. In constructing a predication these rules identify predicates and are then supported by argument identification rules. Such rules apply several constraints: No argument can be used in more than one predication unless it is coordinate or the head of a relative clause. For example, in (9), since *PARP-1 *and *nuclear factor kappa B *are coordinate each is allowed to participate in a separate ASSOCIATED_WITH predication with inflammatory disorders. In (10), *Tag *is the head of relative clause (marked by the following *which*) and is thus allowed to serve as the subject of an INHIBIT relation with *p53 *and also as the subject of an ASSOCIATED_WITH relation with *insulinoma*.

(9) **PARP-1 **and **nuclear factor kappa B **have both been suggested to play a crucial role in inflammatory disorders.

(10) However, **Tag**, which inactivates the key tumour suppressor p53, is not known to be involved in the pathogenesis of human insulinoma.

Subjects appear to the left of an indicator, and objects to the right for active constructions; for passives the order is reversed. *Human obesity syndrome *in (11), for example, will be interpreted as the object of a causes relation, because it occurs to the left of the passive indicator *caused*; *MKKS*, to the right, will be the subject.

(11) We and others have demonstrated that **human obesity syndrome **is caused by mutations in the gene **MKKS**.

**Figure 2:**
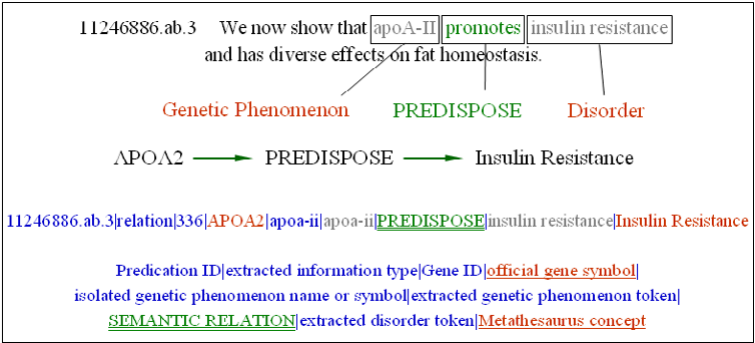
**Example of *SemGen *gene-disease semantic relation and *SemGen *output fields**. *SemGen *output includes semantic relation in green, double underlined; arguments in red, single underlined; extracted genetic phenomenon and disorder tokens in gray.

Finally, arguments are constrained by semantic class: Both arguments of a gene-gene interaction predicate (STIMULATE, INHIBIT, INTERACT_WITH) must be genetic phenomena. The subject of a gene-disorder predicate (ASSOCIATED_WITH, PREDISPOSE, CAUSE) must be a genetic phenomenon, while its object is a disorder. An example of *SemGen *output is given in Figure 2, which provides the final interpretation for the example from (5) above.

### GFINDer

In order to exploit extracted semantic predications, we used the *GFINDer *(Genome Function INtegrated Discoverer) application [12-15]. This is a Web application that enriches lists of gene IDs with controlled functional annotations dynamically retrieved from several biomolecular databanks, including Entrez Gene [16], Gene Ontology [17], KEGG [18], Swiss-Prot [19], Pfam [20], and OMIM [21]. Moreover, *GFINDer *allows computational and statistical analyses on the functional and phenotypic annotations of user-selected groups of genes, aimed at highlighting those annotation categories that are significant in the genes selected.

### Overview

After establishing a baseline for *SemGen *processing on the basis of a corpus of Medline citations on diabetes, we evaluated the results of postprocessing this *SemGen *output with a distance filtering procedure we developed. We also compared these results to those obtained from applying our filtering method to genetic (gene-gene) and etiologic (gene-disease) relations obtained with co-occurrence processing applied to the same corpus on diabetes. Finally, we tested the usefulness of the filtered information obtained from *SemGen *by looking at genes extracted from text on Parkinson's disease and enhanced with annotations using *GFINDer*.

## Results

### Postprocessing

**Figure 3:**
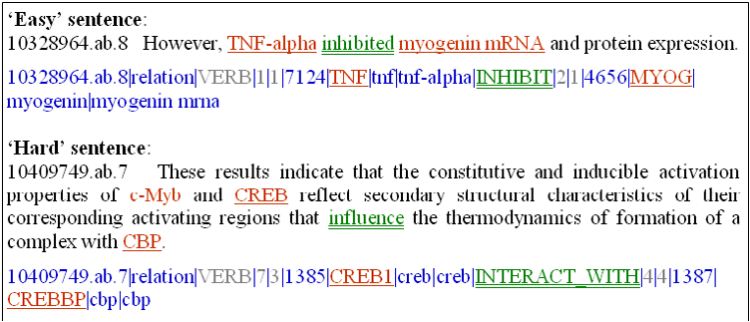
**Examples of 'easy' and 'hard' sentences expressing a gene-gene semantic relation, and their *SemGen *output**. Extracted arguments are shown in red, single underlined. Indicator and corresponding relation are shown in green, double underlined. *SemGen *output includes argument distances and indicator syntactic category, in gray.

The postprocessing procedure for increased accuracy is based on the intuition that syntactic complexity correlates with reliability in NLP. One aspect of complexity is argument proximity to indicator, and anecdotal evidence suggests that arguments close to their indicator (in 'easy' structures) are more likely to be correctly identified by *SemGen*. For example, in Figure 3 the 'easy' sentence has arguments contiguous to the indicator "inhibited," and *SemGen *identifies them correctly. By contrast, in the 'hard' sentence, *SemGen *did not correctly identify the arguments of the predication indicated by "influence" (INTERACT_WITH). Although both "CREB" and "CBP" are of the appropriate semantic class to serve as arguments of this gene-gene interaction predication, they do not act in that capacity in this sentence. Based on examples such as those in Figure 3, we hypothesized that postprocessing based on argument distance could enhance *SemGen *accuracy. We further assumed that syntactic type of indicator (verb or preposition) would have a bearing on reliability, since in addition to proximity, structural aspects such as these are an aspect of complexity.

In order to test this hypothesis, we implemented a procedure that first kept track of indicator category and argument distance in *SemGen *output, and then devised a parametric filtering procedure based on these phenomena. Argument distance was computed as the number of phrases intervening between an argument and its indicator.

We established a baseline by calculating the precision of 2,042 unfiltered *SemGen *relations extracted from text on diabetes, as described in the Methods section. Table 1 provides results. Predications both with and without an official gene symbol and Entrez Gene ID are given, as well as the type of relation (gene-gene or gene-disease) and indicator responsible (verb or preposition). (The Entrez Gene ID is important because it is required for subsequent processing with *GFINDer*.) As noted earlier, no gene-gene relations with a preposition indicator were extracted by *SemGen*. Precision for gene-gene relations in particular is not adequate for subsequent unsupervised processing.

**Figure 4:**
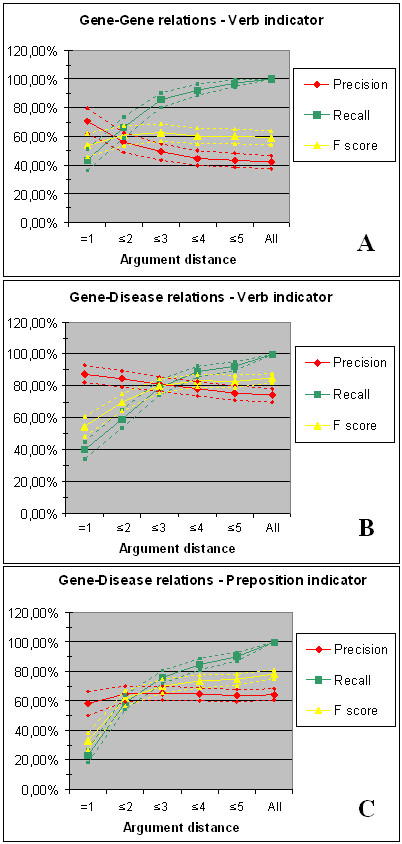
**Filtering semantic relations, with official symbol for each extracted gene, at increasing argument distance**. Argument distance indicates the maximum distance of each argument from the indicator in the relations retained after filtering. "All" refers to no filtering. Precision (*P*), Recall (*R*), and F score (*F*) were calculated as follows: *P *= *rc/(rc+ri)*, *R *= *rc/(rc+dc)*, *F *= 2*P*R/(P+R) with *rc *the retained correct relations, *ri *the retained incorrect relations, and *dc *the discarded correct relations. Dotted lines indicate limits of 95% confidence intervals.

We evaluated the performance of the postprocessing procedure using the same 2,042 relations used to generate *SemGen *baseline figures. Figure 4 shows results (precision, recall, and F score) only for the relations in which all genes have been mapped to the official gene symbol and Entrez Gene ID. Graph A contains figures for gene-gene relations with verb indicators, while Graphs B and C have results for gene-disease relations, B for verb indicators and C for prepositions. "All" refers to no filtering (baseline); values at this point correspond to the relevant line from Table 1 (with official symbol).

The use of this filtering procedure implies an inverse relationship between precision and amount of information retained (recall): as precision increases, more information is lost. In all cases, argument distance correlates with this trade-off. For example, if gene-gene relations from verb indicators are filtered for arguments at distance 1 from the indicator, precision increases from 41.95% (95% confidence intervals 37.17% to 46.73%) (baseline) to 70.75% (95% confidence intervals 62.09% to 79.41%) (Figure 4, Graph A); however, information retained (recall) drops to 43.60% (95% confidence intervals 36.19% to 51.02%).

Results demonstrate that proximity to verb indicator has a positive effect on accuracy. In addition, semantic class of predicate (gene-gene or gene-disease) appears to influence reliability. With regard to type of indicator, precision is higher for gene-disease relations with verb indicator (B) than for the same relations with preposition indicator (C). Although we did not conduct a formal error analysis to explicate this difference, it is likely dependent on the fact that "in" as an indicator is ambiguous, and hence prone to generating *SemGen *errors. Another observation is that for verb indicators, precision is higher for gene-disease relations (B) than for gene-gene relations (A). Again, we can only provide an impressionistic explanation. This result is probably due to the tendency for disorder names to be easier to identify than gene names.

### Proximity in co-occurrence processing

**Figure 5:**
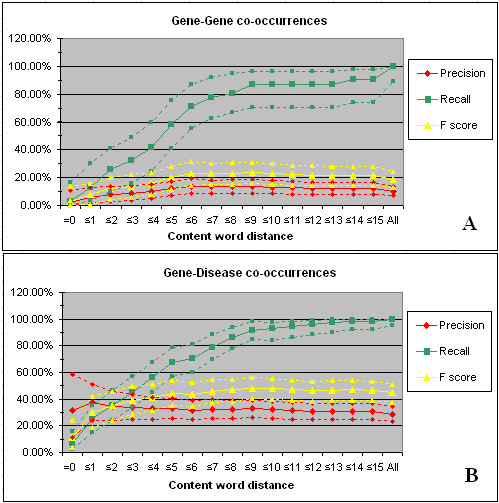
**Filtering relations between co-occurring gene-gene or gene-disease terms at increasing number of intervening content words**. Content word distance indicates the maximum distance in number of intervening meaningful words between two cooccurring gene-gene or gene-disease terms in the relations retained after filtering. "All" refers to no filtering. Precision (*P*), Recall (*R*), and F score (*F*) were calculated as follows: *P *= *rc/(rc+ri)*, *R *= *rc/(rc+dc)*, *F *= 2*P*R/(P+R) with *rc *the retained correct relations, *ri *the retained incorrect relations, and *dc *the discarded correct relations. Dotted lines indicate limits of 95% confidence intervals.

To assess the generality of distance based filtering, we applied this postprocessing to gene-gene and gene-disease relations identified with co-occurrence processing. After using *SemGen *to identify relevant entities in the Medline citations on diabetes noted above, we computed gene-gene and gene-disorder co-occurrence, and then computed the distance (in content words) between each co-occurrence pair. The results are shown in Figure 5. "All" indicates no filtering, and the other points denote "bins" of cumulative intervening content words ("≤ 10" covers 0 to 10 content words, for example).

Results show that precision varies with distance, reaching best values for moderate to -intermediate distances for gene-gene co-occurrences and low distances for gene-disorder co-occurrence pairs. Filtering at such distances improves results when compared to no filtering. For gene-disease co-occurrences, precision improved by 30.46% when considering only co-occurrences with no more than 1 intervening content word: 37.50% vs. 28.74% precision (95% confidence intervals 23.80% to 51.20% vs. 23.10% to 34.39%). Whereas, for gene-gene co-occurrences improvement when considering only co-occurrences with up to 6 intervening content words was 32.62%: 13.75% vs. 10.37% precision (95% confidence intervals 8.41% to 19.09% vs. 6.91% to 13.82%). When the number of intervening content words is low (up to 4), precision is decreased for gene-gene co-occurrences.

The effect of proximity filtering on overall precision of co-occurrence processing is not dramatic. In addition, the improved precision results have a wide margin that partially overlaps with the unfiltered results, suggesting that in some cases the improvement could be due to variation. However, these results do not limit the benefits of such filtering for semantic interpretation. As noted above, the effectiveness of proximity processing with semantic interpretation is ultimately determined by the structure of English sentences. Co-occurrence processing is applied without reference to that structure, and hence subsequent proximity processing either has minimal effect or depresses precision (with fewest intervening content words).

### Exploiting extracted relations

**Figure 6:**
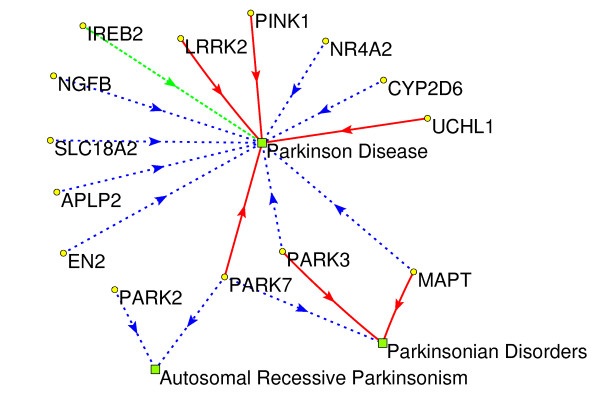
**Etiologic semantic relations regarding Parkinson's disease extracted with NLP and filtered with the postprocessing procedure**. Yellow circles represent genes. Green squares symbolize diseases. Arrowed lines in red (solid), light green (densely dotted), and blue (lightly dotted) represent CAUSE, PREDISPOSE, and ASSOCIATED_WITH semantic relations, respectively.

As noted above, we used text on the molecular genetics of diabetes to evaluate the accuracy of our postprocessing filter. We also conducted a second test to demonstrate the usefulness of filtered predications for subsequent unsupervised processing. For this, we focused on Parkinson's disease, whose polygenetic etiology is not fully understood [22-24]. Eighty-four etiologic relations on Parkinson's related disorders containing an official gene symbol and Entrez Gene ID were extracted with *SemGen *from Medline citations on Parkinson's disease. These were then subjected to postprocessing limited to verb indicators and a maximum argument distance of 3. The 18 distinct remaining relations contained 14 unique genes (Figure 6). Five of these (APLP2, EN2, IREB2, NGFB, SLC18A2) were not associated with Parkinson's disease or related disorders in several important genetic disorder databases, including the Online Mendelian Inheritance in Man (OMIM), Genetics Home Reference (GHR) [25], Genetic Association Database (GAD) [26], or the Parkinson Disease mutation database (MutPD) [27].

**Figure 7:**
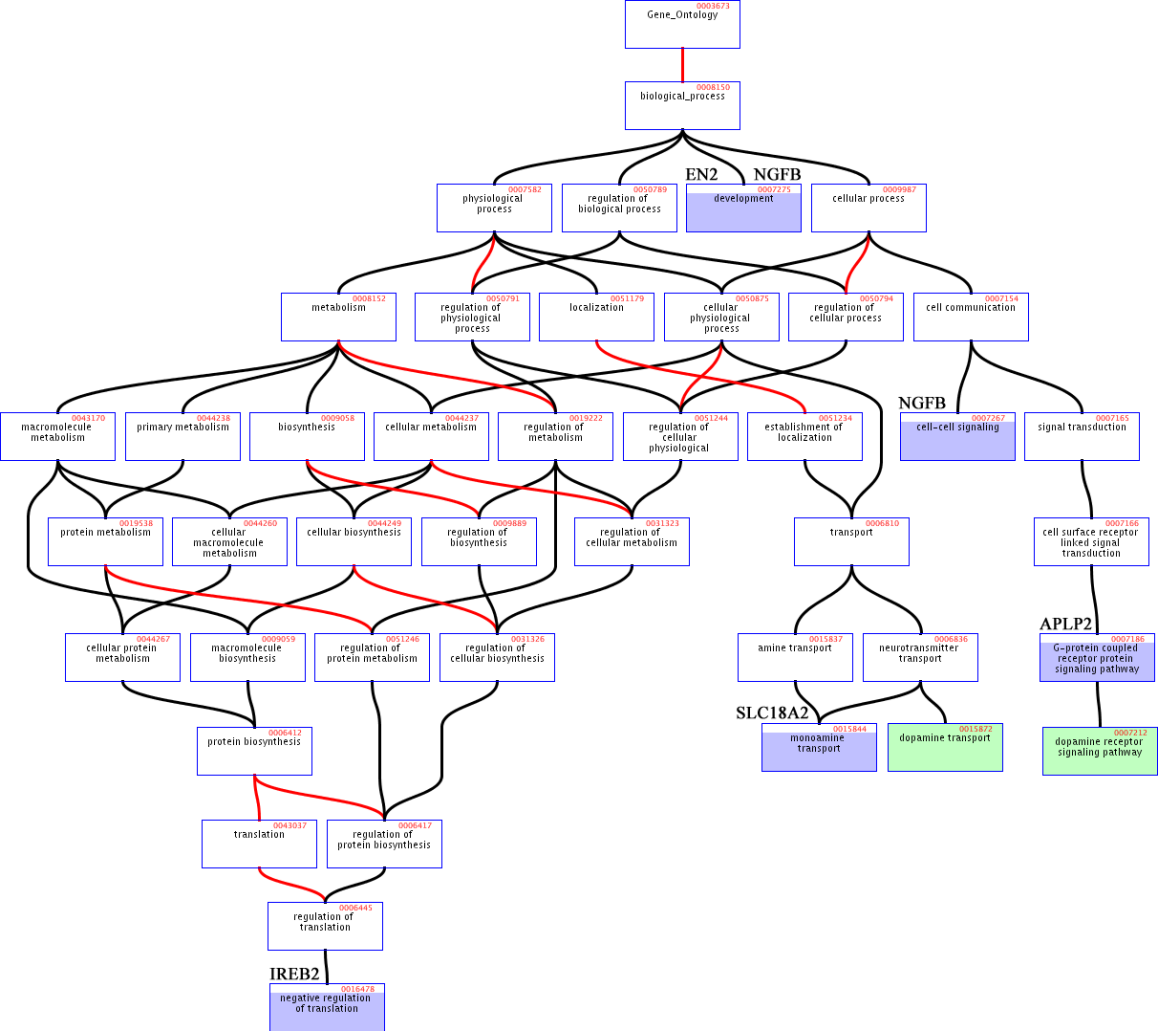
**Gene Ontology biological process categories associated with the five genes selected, but not present in reference databases, as related to Parkinson's disease**. Boxes in dark purple represent the most specific biological process categories the selected five genes have been annotated with (associated gene symbol above the box). Because of inheritance in the Gene Ontology hierarchy, genes are also annotated with all parent categories. Boxes in light green denote dopamine biological process categories that are direct child or sibling of two of the most specific biological process categories associated with two of the selected five genes. Note that motor symptoms in Parkinson's disease are generally thought to result from deficiency or dysfunction of dopamine or dopaminergic neurons in the substantia nigra [22]. Box category links in dark black represent IS-A relations, whereas those in light red represent PART_OF.

To obtain more information on those five genes, we loaded their Entrez Gene IDs into *GFINDer*, which enabled us to find the biological process categories in the Gene Ontology that were assigned to the selected genes. We did not take into account annotations inferred from electronic annotation and those with negative evidence. The annotations used, shown in Figure 7, are mainly related to cellular metabolism and its regulation, cell communication, and transport. In particular, they include "development" (assigned to EN2 and NGFB); "cell-cell signaling" (NGFB); "negative regulation of translation" (IREB2); "G-protein coupled receptor protein signaling pathway" (APLP2), which is a parent of "dopamine receptor signaling pathway"; and "monoamine transport" (SLC18A2), a sibling of "dopamine transport." Note that because of inheritance in the Gene Ontology hierarchy, all ancestor categories are also assigned to these genes.

**Figure 8:**
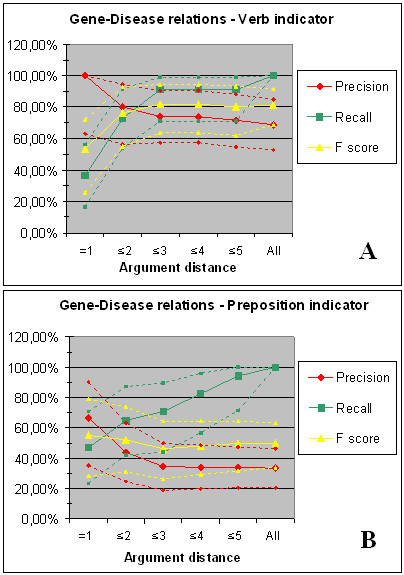
**Parkinson-related etiologic relations, with official symbol for each extracted gene, filtered at increasing argument distance**. Argument distance indicates the maximum distance of each argument from the indicator in the relations retained after filtering. "All" refers to no filtering. Precision (*P*), Recall (*R*), and F score (*F*) were calculated as follows: *P = rc/(rc+ri)*, *R = rc/(rc+dc)*, F = 2*P*R/(P+R) with *rc *the retained correct relations, *ri *the retained incorrect relations, and *dc *the discarded correct relations. Dotted lines indicate limits of 95% confidence intervals.

We also manually assessed the correctness of the 84 relations by comparing them to the original sentences. We then calculated precision before and after filtering, and recall and F score after filtering. Figure 8 shows the results for verb and preposition indicators at several argument distance values. They illustrate that by considering only relations with verb indicators (Graph A) and maximum argument distance of 3, we obtained both high precision (74.07%, 95% confidence intervals 57.54% to 90.60%) and recall (90.91%, 95% confidence intervals 70.84% to 98.88%) for the selected relations. The breadth of the confidence intervals is due to the small number of semantic relations assessed.

## Discussion

### NLP to support research in molecular biology

*SemGen *is one of several systems currently being developed to provide access to information in text (entities and relations between them) for molecular biology research (see [28] for an overview). Of the systems that identify relations, various approaches (both statistical and rule based) are being pursued. Due to the complexity of the content involved, most systems focus on a particular molecular biology phenomenon. Several applications address protein-protein interactions: Bunescu et al. [29], for example, use machine learning techniques, while Corney et al. [30] employ syntactic patterns for such relations. Blaschke et al. [31] and Blaschke & Valencia [32] also use syntactic templates, enhanced with proximity processing between arguments, to identify protein interactions. Huang et al. [33] also call on syntactic patterns, which they discover with automatic methods, and Temkin and Gilder [34] use a context free grammar to extract protein interactions from text. Hu et al. [35] concentrate on protein phosphorylation using a system similar to *SemGen*. Regarding function and structure, Gaizauskas et al. [36] employ both syntactic parsing and semantic templates to identify information about protein structure in text. Koike et al. [37] exploit syntactic patterns for gene function, and Daraselia et al. [38] take advantage of a full parse for protein function. Friedman et al. [39] use a semantic grammar to identify molecular pathways, while Santos et al. [40] combine statistical methods with partial and full parsing and concentrate on the Wnt pathway. Blaschke et al. [41] mine gene expression information from Medline citations using a method similar to [31,32]. Finally, Leroy et al. [42] exploit a shallow parser to identify various relations in molecular biology. Proximity between arguments is also used in their method.

The postprocessing technique we developed selects the extracted semantic relations that are most likely to be correct based on distance of the arguments from the syntactic predicate (indicator). Other methods [31,32,41,42] have employed a related notion, namely distance between arguments participating in a relation, where the relations are identified with templates or shallow parsing. Previous work has not discussed incremental improvements dependent on degree of proximity, nor discussed the recall-precision trade-off, nor compared proximity filtering to unfiltered results.

Although we based our work on *SemGen*, our filtering process could be applied to relations produced by other NLP methods. The semantic content of the relations is not relevant. The postprocessing technique could be transferred most straightforwardly to those systems that retrieve arguments using rules or patterns, since a verb or preposition (an indicator) is available to interact with argument distance as a predictor of extraction accuracy. When an indicator is not used, as in statistical systems, the technique could be slightly modified to use distance between arguments as the sole predictor of correctness. However, in this case it is not likely to be dramatically effective, as suggested by our experiment with co-occurrence processing.

### Argument distance thresholds while postprocessing extracted relations

Effectively exploiting extracted genetic and etiologic relations for subsequent applications depends on maintaining a balance between the highest possible precision and a sufficient level of retained information (Figure 4) for useful applications. For gene-gene relations derived from verbs, for example, this can be obtained by allowing an argument distance of no more than 2 phrases from the indicator (55.88% (95% confidence intervals 49.07% to 62.70%) precision, and 66.28% (95% confidence intervals 59.21% to 73.34%) recall). However, when filtered relations are used for automatic processing, high precision should take precedence over high recall (retained information): for verb indicators, an argument distance of 1 for genetic relations and 2 or 3 for etiologic relations. For supervised applications less strict threshold values can be used.

Considering all extracted relations, whether they include an official gene symbol or not, would increase precision for any distance threshold (Table 1). Such relations could be useful for subsequent supervised applications; however, we limited this study to official gene symbols (and the corresponding Entrez Gene ID) because this allows automatic linking to structured biological data. Subsequent automatic processing based on this information could then unveil hidden biological knowledge [43-45].

### Exploiting extracted information

The application example discussed above illustrates that the procedure we propose for filtering the results of automatically extracted gene-gene and gene-disease relations effectively selects useful information. Many of the genes identified, including CYP2D6, LRRK2, MAPT, NR4A2, PARK2, PARK3, PARK7, PINK1, UCHL1 (Figure 6), are associated with Parkinson's disorders in genetic disorder reference databases, such as OMIM, GHR, GAD, and MutPD. Furthermore, the process identified five genes not associated with Parkinson's disease in those databases (APLP2, EN2, IREB2, NGFB, and SLC18A2). By uploading these genes into *GFINDer*, we were able to highlight their biological process categories in the Gene Ontology (Figure 7). Three of these genes (EN2, IREB2, and NGFB) are currently associated only with high level or general biological process categories that might or might not be related to Parkinson's disease. However, two of these genes (APLP2 and SLC18A2) are associated with low level biological process categories clearly related to Parkinson' disease: "G-protein coupled receptor protein signaling pathway" and "monoamine transport", respectively. Alterations in the biological processes of these categories, which are parent and sibling categories of "dopamine receptor signaling pathway" and "dopamine transport" respectively, may well be involved in Parkinson's disease and suggest interesting avenues for further analysis. In fact, motor symptoms in Parkinson's disease are generally thought to result from deficiency or dysfunction of dopamine or dopaminergic neurons in the substantia nigra [22].

### Future work

Although current filtering usefully supports subsequent automatic analysis, there is room for improvement. As a further measure of sentence complexity, an extended multi-parametric filtering could be implemented, which takes into account the total number of arguments on both the left and the right of an indicator. It would also be possible to improve results by exploiting domain knowledge about genes and diseases to support statistical methods for constructing resources expressing functional characteristics such as involvement in biological processes, biochemical pathways, molecular functions, and co-occurring expression in similar tissues. This information could then be consulted to exclude improbable semantic relations.

## Conclusion

The genetic and etiologic relations extracted by *SemGen *from the research literature are normalized semantic descriptions of complex genetic interactions. The filtering method we implemented increases the precision of error-prone NLP by selecting the semantic relations most likely to be correct. This information can then be used for further applications aimed at uncovering new biomedical knowledge.

## Methods

### Establishing a baseline

To establish a baseline, we first evaluated the precision of *SemGen *in extracting genetic (gene-gene) and etiologic (gene-disease) relations from 5,525 Medline citations on the genetic basis of diabetes retrieved with the PubMed query "diabetes AND (gene OR genes OR genetic)." From these citations *SemGen *extracted a total of 8,956 genetic and etiologic relations. 2,042 (22.80%) of them were selected from 1,934 sentences and were compared to the original sentences by a genetics domain expert, who classified them as correct or not. The primary consideration in selecting the relations to evaluate was whether all gene names in the relation had been matched to the official gene symbol (in Entrez Gene). The official symbol is required by *GFINDer *to connect information extracted by *SemGen *to online resources. 1,372 (of the 8,956 total relations) had gene names matched to Entrez Gene and all such relations were evaluated. Of these, 410 referred to gene-gene relations and 962 to gene-disease predications. 817 relations with an official gene symbol had been derived from a verb indicator by *SemGen*, and 555 from a preposition. In addition, 670 extracted relations without an official gene symbol and Entrez Gene ID were also randomly selected and assessed (617 gene-gene and 53 gene-disease relations; 643 with verb indicator and 27 with preposition indicator). The results of the evaluation for the 2,042 total semantic relations assessed (1,027 gene-gene and 1,015 gene-disease relations; 1,460 with verb indicator and 582 with preposition indicator) are reported in Table 1.

### Filtering *SemGen *output

As a gold standard for evaluating the filtering strategy to retain the relations most likely to be correct, we used the same 2,042 semantic relations about diabetes on which the baseline was determined. We filtered the gold standard relations at various thresholds of argument distance from the relation indicator by keeping all relations with both argument distances lower or equal to the considered threshold. After each filtering, we grouped the selected relations according to relation type (gene-gene or gene-disease), syntactic category of indicator (preposition or verb), and official gene symbol content (relations with official gene symbol for all extracted genes or not for all). For each group we calculated number of correct and incorrect relations, precision (*P*) (the percent of semantic relations retained after filtering that are correct), recall (*R*) or retained information (the percent of the initially correct semantic relations retained after filtering), and F score (*F*) (the harmonic mean of precision and recall) as follows: *P = rc/(rc+ri)*, *R = rc/(rc+dc)*, F = 2*P*R/(P+R) with *rc *the retained correct relations, *ri *the retained incorrect relations, and *dc *the discarded correct relations. 95% confidence intervals were also computed for each calculated value of *P*, *R*, and *F*.

### Co-occurrence processing

We conducted co-occurrence processing on the same text used to evaluate postprocessing of *SemGen *predications (the 5,525 Medline citations on the genetic basis of diabetes discussed above). After using *SemGen *to identify genetic phenomena and disorders (but not predications) in this text, we computed the number of content words that intervened between all co-occurrences of gene-gene and gene-disorder concepts. Content words were considered to be numbers (whether expressed as digits or words), adverbs, adjectives, nouns, and all verb forms (e.g. *cause, causes, caused*, and *causing*). For example, *SemGen *identified a genetic phenomenon concept (*DJ-1*) and a disorder (*early-onset autosomal recessive Parkinson's disease*) in (12). The distance between them is two content words (the noun *gene *and the verb form *cause*).

(12) Mutations in the *DJ-1 *gene cause *early-onset autosomal recessive Parkinson's disease*

In evaluating the co-occurrence processing, a sample of 200 sentences (10.34%) was randomly selected from the same 1,934 sentences in the Medline citations on diabetes used to evaluate proximity filtering based on *SemGen *processing. Then, a domain expert assessed 546 co-occurrences (299 gene-gene and 247 gene-disease) within these 200 sentences and classified them as correct or not.

We subsequently evaluated the effectiveness of distance filtering postprocessing to improve precision of extracted co-occurrences. We first filtered the assessed co-occurrences at incremental distance thresholds of intervening content words between co-occurrences of gene-gene or gene-disorder concepts. Then, as was done for evaluating *SemGen *output filtering, for each threshold value we calculated the number of correct and incorrect relations, and precision, recall, F score and 95% confidence intervals.

### Processing for exploiting extracted relations

A PubMed query on the genetics of Parkinson's disease retrieved 3,871 Medline citations. Initial *SemGen *processing extracted 1,454 semantic relations from 899 citations. Of these, 365 were gene-gene relations (all with verb indicator; 40 with an official gene symbol and 325 without), and 1,089 were gene-disease relations (454 with verb indicator, 635 with preposition indicator; 233 with an official gene symbol and 856 without). For further processing, we limited the extracted relations to the 85 etiologic relations which included an official gene symbol and Entrez Gene ID and which involved one of the following disorders: Parkinson Disease (74 relations), Parkinsonian Disorders (9), and Autosomal Recessive Parkinsonism (2). One of these relations was subsequently eliminated because it negated a gene-disease association. We then evaluated the performance of distance filtering on these 84 semantic relations comparing them against the sentences that generated them, filtering them at increasing distance thresholds, and calculating precisions, recalls, F scores and their 95% confidence intervals.

## Authors' contributions

MM conceived of the study, performed the analyses, and drafted the manuscript. HK was responsible for informatics development. FML conducted the co-occurrence processing. TCR participated in the design and coordination of the study and helped draft the manuscript. All authors read and approved the final manuscript.

## Figures and Tables

**Table 1:** *SemGen *precision for 2,042 genetic (gene-gene) and etiologic (gene-disease) semantic relations

**Semantic relations**	**Syntactic indicator**	**Official symbol**	**Total relations**	**Correct relations**	**Incorrect relations**	**Precision**	**Standard error**
gene-gene	verb	yes	410	172	238	41.95%	2.44%
gene-gene	verb	no	617	299	318	48.46%	2.01%
gene-disease	verb	yes	407	302	105	74.20%	2.17%
gene-disease	verb	no	26	21	5	80.77%	7.73%
gene-disease	preposition	yes	555	357	198	64.32%	2.03%
gene-disease	preposition	no	27	17	10	62.96%	9.29%

"Official symbol" indicates whether all relevant relations include an official gene symbol and Entrez Gene ID for each extracted gene or not.
